# Determination of available and total tract digestible phosphorus release curves for Microtech phytase

**DOI:** 10.1093/tas/txag006

**Published:** 2026-01-14

**Authors:** Ron Aldwin S Navales, Katelyn N Gaffield, Ty H Kim, Joel M DeRouchey, Paul Martin, Juan A Javierre, Jordan T Gebhardt, Robert D Goodband, Mike D Tokach, Jason C Woodworth

**Affiliations:** Department of Animal Sciences and Industry, College of Agriculture, Kansas State University, Manhattan, KS, 66506-0201, United States; Department of Animal Sciences and Industry, College of Agriculture, Kansas State University, Manhattan, KS, 66506-0201, United States; Department of Animal Sciences and Industry, College of Agriculture, Kansas State University, Manhattan, KS, 66506-0201, United States; Department of Animal Sciences and Industry, College of Agriculture, Kansas State University, Manhattan, KS, 66506-0201, United States; Imogene Ingredients, West Des Moines, IA, 50265, United States; JA Santafe Venture S.L., Barcelona, 08480, Spain; Department of Diagnostic Medicine/Pathobiology, College of Veterinary Medicine, Kansas State University, Manhattan, KS, 66506-0201, United States; Department of Animal Sciences and Industry, College of Agriculture, Kansas State University, Manhattan, KS, 66506-0201, United States; Department of Animal Sciences and Industry, College of Agriculture, Kansas State University, Manhattan, KS, 66506-0201, United States; Department of Animal Sciences and Industry, College of Agriculture, Kansas State University, Manhattan, KS, 66506-0201, United States

**Keywords:** available phosphorus, bone ash, nursery pigs, phytase, total tract digestible phosphorus

## Abstract

A total of 320 barrows (DNA 200 × 400; initially 10.7 ± 0.21 kg) were used in a 21-d growth trial to determine the available P (aP) and true total tract digestible P (TTTD P) release curves for Microtech phytase (VTR Bio-Tech Co, Ltd, Guangdong, China). At ∼21 d of age, pigs were weaned, randomly allocated to pens and fed a common diet for 18 d and then fed a P deficient diet with 0.11% aP and 0.20% standardized total tract digestible (STTD P) for 3 d. On d 21 post-weaning, considered d 0 of the study, pigs were blocked by average pen body weight (BW) and randomly allotted to 1 of 8 dietary treatments with 5 pigs per pen and 8 pens per treatment. Dietary treatments were derived from a single basal diet and phytase, monocalcium phosphate, limestone, and sand were added to create the treatment diets. The basal diet was formulated to contain 0.32% phytate P. Treatments included 3 diets with 0.11%, 0.19% and 0.27% aP (corresponding to STTD P of 0.20%, 0.27% and 0.35%) using monocalcium phosphate and 5 diets with 0.11% aP (0.20% STTD P) and increasing phytase (250, 500, 1000, 1500 and 2000 FTU/kg). On d 14 of the trial, fecal samples were collected from 3 pigs per pen. Samples were analyzed for dry matter (DM), N, P, and TiO_2_ for the calculation of apparent total tract digestibility (ATTD) of DM, N and P. At the conclusion of the experiment, 1 pig, closest to the mean weight of each pen, was euthanized for bone analysis. The right fibula, 10th rib, and third metacarpal were collected for the determination of bone density, bone ash weight, and percentage bone ash. Increasing aP and STTD P from inorganic P increased (quadratic, *P ≤ *0.050) final BW, average daily gain (ADG), and gain to feed ratio (G:F). Increasing phytase increased (linear, *P ≤ *0.012) final BW and average daily feed intake (ADFI) and improved (quadratic, *P ≤ *0.042) ADG and G:F. Dry matter digestibility decreased (quadratic, *P = *0.043) with increasing aP and STTD P from inorganic P. Phosphorus digestibility increased (linear, *P ≤ *0.001) with increasing aP and STTD P from inorganic P and increasing phytase. Bone density, bone ash weight, and percentage bone ash increased (linear, *P ≤ *0.004) with increasing aP and STTD P from inorganic P and increasing phytase. The aP release curve based on the average percentage bone ash of the three bones and TTTD P release curve developed for Microtech phytase are: aP release, % = (0.868 × FTU/kg) ÷ (9599.511 + FTU/kg) and TTTD P release, % = (0.00004017 × FTU) + 0.011, respectively.

## Teaser

The experiment generates release curves for available and digestible P using a commercially available phytase source.

## Introduction

Phytase (*myo*-inositol hexaphosphate phosphohydrolase) is an enzyme used to hydrolyze the phytate molecule and release phytate P (Selle and Ravindran 2008). The use of phytase in animal diets allows for greater digestion of P and other nutrients in plant-based ingredients, thereby improving animal performance while reducing P excretion. In swine nutrition, phytase can be sourced from endogenous enzymes generated by the small intestine mucosa, microfloral enzyme activity in the large intestine, intrinsic plant phytase activity, and dietary inclusion of exogenous phytase. Available P (aP) release by phytase is determined based on its biological value relative to a standard mineral source such as monocalcium phosphate using a sensitive response indicator such as bone mineralization as response criteria (Zhai et al. 2022). Available P release can then be converted to digestible P release using a conversion factor of 0.883 (NRC 1998). Improvement in P digestibility by phytase can also be measured by comparing the amount of digestible P in P-deficient diets with and without phytase (Almeida et al. 2013). Digestible P can be expressed as apparent total tract digestible P (ATTD P), standardized total tract digestible P (STTD P) or true total tract digestible P (TTTD P). Apparent total tract digestible P measures the proportion of P that is not excreted in the feces without correcting for endogenous losses, whereas STTD P and TTTD P corrects for basal and total endogenous losses, respectively.

Microtech phytase is a new generation phytase developed by VTR Biotech (Guangdong, China) with little data available to describe its P release. Microtech phytase is an *E.* coli-derived 6-phytase expressed in *Pichia pastoris*. A study by Flohr et al. (2015) observed that inclusion of Microtech phytase resulted in improved average daily gain (ADG), feed to gain ratio, and bone ash weight and percentage up to its highest inclusion in the study of 780 FTU/kg indicating that higher inclusion may provide additional benefit to growth performance and bone mineralization. Flohr et al. (2015) found that aP release of Microtech phytase ranged from 0.02% to 0.06% when included at 155 to 780 FTU/kg. Evaluation of Microtech phytase warrants a higher inclusion rate and development of both aP and digestible P release. Therefore, the objectives of the study were to evaluate the effects of Microtech phytase on growth performance, fecal P digestibility, and bone characteristics of 10- to 22 kg nursery pigs and to develop available and TTTD P release curves for Microtech phytase at levels between 250 to 2000 FTU/kg.

## Materials and methods

The protocol used in this experiment was approved by the Kansas State University Institutional Animal Care and Use Committee. Prior to the experiment, two samples of corn, soybean meal, canola meal, limestone, monocalcium phosphate, vitamin premix, and mineral premix were analyzed in duplicate for Ca and P (AOAC 2006, official method 985.01) at Kansas State University Research and Extension Soil Testing Laboratory (Manhattan, KS) and Midwest Laboratories (Omaha, NE). The mean values obtained from the two laboratories were used in diet formulation (Table 1). Additionally, three samples of Microtech phytase were analyzed in duplicate (AOAC 2000, official method 2000.12) for phytase activity. Microtech phytase was found to contain 10,758,000 phytase units (FTU)/kg and this was used to determine its inclusion rate in diet formulations.

**Table 1.** Analyzed composition of ingredients used in diet formulation (as-fed basis).

Ingredient	**Ca, %** ^a^	**P, %** ^a^	**Phytase, FTU/kg** ^b^
**Corn**	0.08	0.31	…
**Soybean meal**	0.63	0.71	…
**Canola meal**	1.56	0.80	…
**Limestone**	38.80	0.01	…
**Monocalcium phosphate**	17.15	21.37	…
**Vitamin premix**	26.17	0.03	…
**Mineral premix**	18.44	<0.01	…
**Microtech phytase**	…	…	10,758,000

a Ca and P levels of ingredients were analyzed at K-State Research and Extension Soil Testing Laboratory, Manhattan, KS and Midwest Laboratories, Omaha, NE. Two samples were sent to each laboratory and each analyzed in duplicate. Values represent the mean of the results obtained from the two laboratories.

b Analyzed at Eurofins Nutrition Analysis Center, Des Moines, IA.

### Diet formulation and manufacturing

A single corn-soybean meal-canola meal-based diet was used to derive dietary treatments. The basal diet was formulated to contain 1.25% SID Lys with all other essential amino acids (AA) set to meet or exceed NRC (2012) requirement estimates as a ratio relative to SID Lys (Table 2). Canola meal was added at 7.6% of the basal diet to achieve the formulated phytate P level of 0.32% and ensure adequacy of substrate for phytase activity. Ingredients including Microtech phytase, monocalcium phosphate, limestone, and sand were added to the basal diet to create the experimental diets (Table 3). A ratio of 1.10:1 analyzed Ca to analyzed P ratio was maintained in all treatment diets. Titanium dioxide (TiO_2_) was added (0.5%) to the diets as an indigestible marker for measurement of nutrient digestibility. Basal and experimental diets were manufactured in meal form at O.H. Kruse Feed Technology Innovation Center at Kansas State University. During bagging, diet samples were collected from every fourth bag, pooled, ground, and stored at −20°C until submitted for analysis. Two samples of each treatment diet were submitted and analyzed in duplicate for Ca and P (Kansas State University Research and Extension Soil Testing Laboratory, Manhattan, KS), phytase activity (AOAC 2000 official method 2000.12), and phytic acid (Ellis et al. 1977; Eurofins Nutrition Analysis Center, Des Moines, IA).

**Table 2.** Composition of basal mix (as-fed basis)^a^.

Item	
**Ingredient, %**	
** Corn**	60.59
** Soybean meal**	29.86
** Canola meal**	7.59
** Sodium chloride**	0.53
** L-Lysine HCl**	0.30
** DL-Methionine**	0.10
** L-Threonine**	0.10
** L-Valine**	0.01
** Trace mineral premix^b^**	0.15
** Vitamin premix^c^**	0.25
** Titanium dioxide**	0.51
**Total**	100.00
**Calculated analysis**	
** Standardized ileal digestible (SID) amino acids**	
** Lys, %**	1.25
** Ile:Lys**	63
** Leu:Lys**	129
** Met:Lys**	33
** Met and Cys:Lys**	59
** Thr:Lys**	63
** Trp:Lys**	18.6
** Val:Lys**	70
** His:Lys**	42
** Total Lys, %**	1.44
** Metabolizable energy, kcal/kg**	3309
** Net energy (NE), kcal/kg**	2416
** SID Lys:NE, g/Mcal**	5.17
** Crude protein, %**	22.6
** Ca, %**	0.45
** P, %**	0.46
** Available P, %^d^**	0.09
** STTD P, %^e^**	0.19

a The basal batch was used as a major ingredient in each experimental diet.

b Provided per kilogram of diet: 110 mg Zn from zinc sulfate; 110 mg Fe from ferrous sulfate, 33 mg Mn from manganese oxide; 17 mg Cu from copper sulfate; 0.30 mg I from calcium iodate; 0.30 mg Se from sodium selenite.

c Provided per kilogram of diet: 4134 IU vitamin A; 1653 IU vitamin D; 44 IU vitamin E; 3 mg vitamin K; 0.03 mg vitamin B_12_, 50 mg niacin; 28 mg pantothenic acid; 8 mg riboflavin.

d Coefficients for formulation were derived from NRC (1998).

e STTD P, standardized total tract digestible phosphorus.

**Table 3.** Composition, calculated analysis, and analyzed composition of experimental diets (as-fed basis).

Item	Inorganic P			**Phytase** ^a^				
	0.11	0.19	0.27	250	500	1000	1500	2000
**Ingredient, %**								
** Basal mix**	98.88	98.88	98.88	98.88	98.88	98.88	98.88	98.88
** Limestone**	0.16	0.23	0.29	0.16	0.16	0.16	0.16	0.16
** Monocalcium P**	0.11	0.49	0.86	0.11	0.11	0.11	0.11	0.11
** Sand^b^**	0.85	0.43	…	0.85	0.85	0.84	0.84	0.83
** Phytase^c^**	…	…	…	0.0023	0.0046	0.0093	0.0139	0.0186
** Total**	100	100	100	100	100	100	100	100
**Calculated analysis**								
** NE, kcal/kg**	2377	2377	2377	2377	2377	2377	2377	2377
** SID Lys, %**	1.24	1.24	1.24	1.24	1.24	1.24	1.24	1.24
** Ca, %**	0.53	0.62	0.70	0.53	0.53	0.53	0.53	0.53
** P, %**	0.48	0.56	0.64	0.48	0.48	0.48	0.48	0.48
** Ca:P ratio**	1.10	1.10	1.10	1.10	1.10	1.10	1.10	1.10
** Phytate P, %**	0.32	0.32	0.32	0.32	0.32	0.32	0.32	0.32
**Analyzed composition^d^**								
** DM, %**	88.33	88.22	88.42	88.51	88.54	88.62	88.60	88.69
** N, %**	3.23	3.22	3.26	3.28	3.64	3.27	3.22	3.31
** Ca, %**	0.46	0.54	0.64	0.46	0.46	0.53	0.47	0.49
** P, %**	0.38	0.46	0.56	0.40	0.42	0.42	0.44	0.45
** Ca:P ratio**	1.21	1.17	1.14	1.15	1.10	1.26	1.07	1.09
** Phytate P, %**	0.28	0.28	0.30	0.27	0.28	0.29	0.29	0.30
** Phytase, FTU/g**	…	…	…	268	483	1060	1575	2225
** TiO_2_**	0.49	0.52	0.51	0.51	0.51	0.52	0.51	0.51

a Microtech phytase, VTR Biotech, Guangdong, China.

b Sand was used to equalize weight of hand-add batch including the addition of limestone, monocalcium P, and phytase when blended with the basal mix.

c Phytase was analyzed for phytase level and contained 10,758,000 FTU/kg (Eurofins Scientific Inc., Des Moines, IA).

d Diet samples were collected from every fourth bag, pooled, ground, and stored at −20°C. Samples were submitted in duplicate at K-State Research and Extension Soil Testing Laboratory, Manhattan, KS for Ca, and P analyses, and at Eurofins Nutrition Analysis Center, Des Moines, IA for phytase and phytic acid analyses. Dry matter, N and TiO_2_ were analyzed at KSU Swine Laboratory. Manhattan, KS.

### Animals and diets

The study was conducted at the Kansas State University Segregated Early Weaning facility in Manhattan, KS. The facility has two identical barns that are completely enclosed and environmentally controlled. Pigs were placed in 1.2 × 1.2 m pens that contained a four-hole dry self-feeder and a cup waterer for ad libitum access to feed and water. A total of 320 barrows (DNA 200 × 400) were weaned at approximately 21 d of age. Weaned pigs were then fed a common diet for 18 d. Pigs were then fed a P deficient diet (0.11% available P corresponding to 0.20% STTDP) for a 3-d period. Then, on the fourth day, pens of pigs were blocked by body weight (BW; initially 10.7 ± 0.21 kg) and randomly allotted to 1 of 8 dietary treatments with 5 pigs per pen and 8 replicate pens per treatment. This was considered d 0 of the study. Treatments included 3 diets with 0.11%, 0.19% and 0.27% aP corresponding to 0.20%, 0.27% and 0.35% STTD P using monocalcium phosphate. These 3 treatment diets were used to develop the standard curves for determining aP and TTTD P release and 5 diets with 0.11% aP (0.20% STTD P) and increasing phytase (250, 500, 1000, 1500 and 2000 FTU/kg) that were used to develop the aP and TTTD P release curves.

### Measurements and sampling

Throughout the 21-d experiment, pig and feeder weights were measured every 7 d to determine ADG, average daily feed intake (ADFI), and gain to feed ratio (G:F). At d 14 of the trial, fecal samples were collected from 3 pigs per pen using rectal stimulation and were stored at −20°C until analysis. Samples were oven dried at 55°C for 48 h, ground, pooled per pen, and analyzed for dry matter (DM), N, P, and TiO_2_ for the calculation of ATTD of DM, N, and P (Equation 1). The weight before and after drying were used to calculate percentage DM. Nitrogen content of the fecal and feed samples was determined by combustion method using Leco TruMac N with TruMac operating software (Leco Corporation, St Joseph, MI), whereas the P content was determined at Midwest Laboratories (Omaha, NE) using inductively coupled plasma spectroscopy-optical emission spectroscopy (AOAC 2006 official method 985.01). Titanium dioxide content of feces and feed were quantified according to Short et al. (1996). Absorbance of standards and samples were read using Epoch 2 plate reader (BioTek Instruments, Inc., Winooski, VT) at 408 nm wavelength. All fecal and feed samples were analyzed in duplicate. For the calculation of ATTD of N and P, the dried fecal samples were further dried in muffle furnace at 135°C for 2 h and this was used to express fecal N, P and TiO_2_ on a DM basis. Furthermore, TTTD of P was calculated based on NRC (2012, Equation 2) with the total endogenous P losses estimated from the *y*-intercept of the regression of ATTD P (g/kg feed) against dietary P concentration (g/kg feed) in inorganic P diets.


(1) $$\text{ATTD}\;P,\;\text{DM}\;\text{or}\;N,\;\%=[1-(\frac{\text{TiO}2\;\text{in}\;\text{diet},\;\%\times \text{DM},\;N\;\text{or}\;P\;\text{of}\;\text{feces},\;\%\;}{\text{TiO}2\;\text{in}\;\text{feces},\;\%\times \text{DM},\;N\;\text{or}\;P\;\text{of}\;\text{diet},\;\%})]\times 100.$$

(2) $$\text{TTTD}\;P,\;\%=\text{ATTD}\;P,\;\%+[(\frac{\text{total}\;\text{endogenous}\;P\;\text{loss}}{P\;\text{in}\;\text{diet}})\times 100].$$

At the termination of the experiment, pigs were euthanized, and bones were collected from the middle weight pig from each pen to determine bone mineralization. The right fibula, 10th rib, and third metacarpal were collected, individually placed in plastic bags with identification, and stored at −20°C until analysis. After freezing, leftover extraneous soft tissue and cartilage caps were removed from each bone. For bone density, bones were submerged in ultra-purified water under vacuum for 4 h. Bones were then weighed while suspended in a vessel of water and the weights were used to calculate bone density. For bone ash, bones were processed using the non-defatted method. Briefly, each bone was dried at 105°C for 7 d in a drying oven and subsequently ashed at 600°C for 24 h in a muffle furnace This method was used to determine total bone ash weight and percentage ash relative to dried bone weight (Wensley et al. 2020).

### Calculations and statistical analysis

Growth performance, digestibility, and bone analyses data were analyzed as a randomized complete block design with pen as the experimental unit, treatment as fixed effect, and weight and barn blocks as random effects. The base model was evaluated using the MIXED procedure of SAS v.9.4 (SAS Institute, Inc., Cary, NC). Linear and quadratic polynomial contrasts were performed to determine the effects of increasing inorganic P and phytase level. Results were considered significant with *P*-values ≤ 0.05 and were considered marginally significant at 0.05 < *P ≤ *0.10.

Available P release by phytase was calculated using a standard response curve that utilized the inorganic P diets. Standard response curves were developed using marginal aP intake (ie, dietary aP% minus 0.11% [the aP in the basal diet] multiplied by ADFI) as a predictor variable for each response criterion. The equation for the standard curve was used to calculate the aP release from each pen based on the observed value for each response criterion. Using the pen ADFI, this value was then converted to a marginal aP intake. A mixed model ANOVA with weight and barn blocks as random effects were used to evaluate aP release as a function of the calculated phytase dosage, assuming an intercept of no aP release for the 0.11% aP diet without phytase. Formulated phytase levels were used to calculate all release values. A model (Equation 3) was fitted to pen release values using non-linear regression. The model parameters were estimated using the nls function from the stat package in R (version 4.3.1, R Core Team, Vienna, Austria) to develop aP release curves for bone density, bone ash weight and percentage bone ash.


(3) $$\text{aP}\;\text{release},\;\%=\;\frac{a\times \text{FTU}}{b+\text{FTU}}.$$

where *a* and -*b* are the horizontal and vertical asymptote, respectively.

The TTTD P release from phytase was calculated using two approaches (Zhai et al. 2023). In the first approach, the aP release values based on percent bone ash were transformed to its equivalent digestible P values by multiplying to the TTTD coefficient of P in monocalcium phosphate that was represented by the slope of the linear regression of ATTD P against the formulated dietary P concentration. In the second approach, TTTD P release by phytase was calculated for each pen by subtracting the average of the TTTD P in control diet from the TTTD P in each diet added with phytase. Models were fitted to pen release values using a linear trendline with the formulated phytase level as fixed effect and weight and barn as random effects. The model parameters were estimated using the lme procedure of R (version 4.3.1, R Core Team, Vienna, Austria) to develop the release curve for TTTD P.

## Results

The analyzed Ca and P of the experimental diets were lower than formulated values but were consistent with increasing Ca and P for the inorganic P treatments (Table 3). Phytase activity of the complete diets increased across phytase treatments with analyzed phytase concentration of 268, 483, 1060, 1575, and 2225 FTU/kg. Phytate P levels of the treatment diets ranged from 0.27% to 0.30%.

Pig fed increasing aP and STTD P from inorganic P had increased (quadratic, *P ≤ *0.050) final BW, ADG, and G:F (Table 4). Pigs fed increasing phytase had increased (linear, *P ≤ *0.012) final BW and ADFI and increased (quadratic, *P ≤ *0.042) ADG and G:F. The calculated aP release values from Microtech phytase based on ADG and G:F also followed a significant quadratic (*P ≤ *0.015, Table 5) pattern.

**Table 4.** Effects of increasing aP (and STTD P) from monocalcium P or Microtech phytase on nursery pig growth performance and bone characteristics^a^^,b^.

										*P* =				
Item	**Inorganic P, % aP** ^c^			**Phytase, FTU/kg** ^d^					SEM	Inorganic P		Phytase P		
	0.11	0.19	0.27	250	500	1000	1500	2000		Linear	Quadratic		Linear	Quadratic
**BW, kg**														
** d 0**	10.7	10.7	10.7	10.7	10.7	10.7	10.7	10.7	0.21	0.801	0.781		0.775	0.759
** d 21**	19.5	20.9	21.1	20.2	20.7	20.9	21.1	21.4	0.31	<0.001	0.050		<0.001	0.063
**d 0 to 21**														
** ADG, g**	402	461	472	435	454	463	472	487	9.6	<0.001	0.043		<0.001	0.042
** ADFI, g**	742	766	760	747	778	762	775	786	13.4	0.325	0.331		0.012	0.671
** G:F, g/kg**	542	602	622	581	584	609	609	619	7.8	<0.001	0.027		<0.001	0.003
**Bone characteristics** ^e^														
**Fibula**														
** Bone density, g/mL**	1.15	1.20	1.21	1.16	1.19	1.18	1.19	1.21	0.014	0.004	0.217		0.001	0.840
** Bone ash, g**	0.640	0.806	0.865	0.666	0.747	0.775	0.873	0.872	0.0325	<0.001	0.164		<0.001	0.199
** Bone ash, %**	42.0	45.3	47.8	40.9	44.0	45.1	45.9	46.6	0.78	<0.001	0.692		<0.001	0.289
**Rib**														
** Bone density, g/mL**	1.17	1.22	1.24	1.18	1.21	1.21	1.22	1.23	0.009	<0.001	0.290		<0.001	0.291
** Bone ash, g**	0.780	1.001	1.182	0.759	0.938	0.990	1.036	1.149	0.0443	<0.001	0.710		<0.001	0.470
** Bone ash, %**	44.5	48.3	50.5	43.8	46.8	48.8	49.1	50.4	0.73	<0.001	0.345		<0.001	0.116
**Metacarpal**														
** Bone density, g/mL**	1.11	1.15	1.15	1.13	1.14	1.14	1.15	1.16	0.006	<0.001	0.081		<0.001	0.482
** Bone ash, g**	0.926	1.091	1.171	1.031	1.054	1.111	1.182	1.215	0.0382	<0.001	0.300		<0.001	0.181
** Bone ash, %**	31.9	34.9	36.3	32.1	34.5	35.1	35.6	37.1	0.84	0.001	0.446		<0.001	0.468
**Average**														
** Bone density, g/mL**	1.15	1.19	1.20	1.16	1.18	1.18	1.18	1.20	0.008	<0.001	0.103		<0.001	0.463
** Bone ash, g**	0.782	0.963	1.073	0.818	0.913	0.959	1.030	1.079	0.0331	<0.001	0.318		<0.001	0.163
** Bone ash, %**	39.5	42.9	44.8	38.9	41.8	43.0	43.5	44.7	0.67	<0.001	0.397		<0.001	0.186
**Digestibility** ^f^														
** Fecal dry matter, %**	26.7	27.3	25.9	27.5	26.0	25.9	28.8	25.1	0.95	0.503	0.344		0.612	0.404
** Fecal N, %**	4.48	4.52	4.47	4.28	4.25	4.25	4.00	4.36	0.108	0.984	0.690		0.141	0.015
** Fecal P, %**	1.91	1.92	2.07	1.77	1.70	1.71	1.70	1.52	0.090	0.220	0.557		0.009	0.965
**DM digestibility, %**	84.14	82.36	83.49	82.78	81.91	82.53	83.81	82.03	0.612	0.427	0.043		0.279	0.493
**N digestibility, %**	79.94	77.39	79.24	79.45	80.71	79.07	81.51	78.15	0.911	0.584	0.049		0.473	0.199
**P digestibility, %**	27.82	32.97	44.31	32.03	33.63	34.39	42.69	44.04	3.268	<0.001	0.412		<0.001	0.924

a A total of 320 pigs (DNA 200 × 400, initially 10.7 ± 0.21 kg) were used in a 21-d growth trial with 5 pigs per pen and 8 replications per treatment.

b ADG = average daily gain; ADFI = average daily feed intake; G:F = gain to feed ratio.

c Inorganic P was added to the diet by increasing monocalcium P.

d Microtech phytase, VTR Biotech, Guagdong, China.

e One pig per pen (8 pigs per treatment) was euthanized and the right fibula, right 10th rib, and right third metacarpal were collected to determine bone density, bone ash weight, and percentage bone ash. After cleaning, bones were submerged in ultra-purified water under vacuum for 4 h. Weights were then collected, and bone density calculated. For bone ash, bones were placed in a drying oven at 105°C for 7 d and then ashed in a muffle furnace at 600°C for 24 h.

f At d 14 of the trial, fecal samples were collected from 3 pigs per pen using rectal swabbing and were stored at −20°C until analysis. Samples were oven dried at 55°C for 48 h, ground, pooled per pen, and analyzed for dry matter (DM), N, P, and TiO_2_ for the calculation of ATTD of DM, N, and P.

**Table 5.** Calculated aP release values (%) based on different response criteria^a^.

Item	Phytase, FTU/kg^b^					SEM	*P* =	
	250	500	1000	1500	2000		Linear	Quadratic
**Performance**								
** ADG**	0.057	0.098	0.119	0.135	0.164	0.0202	<0.001	0.015
** G:F**	0.067	0.069	0.121	0.119	0.137	0.0157	<0.001	0.002
**Bone characteristics** ^c^								
**Fibula**								
** Bone density**	0.004	0.062	0.064	0.090	0.152	0.0428	0.002	0.899
** Bone ash**	0.005	0.067	0.084	0.151	0.147	0.0209	<0.001	0.106
** Bone ash**	−0.034	0.043	0.082	0.102	0.119	0.0221	<0.001	0.299
**Rib**								
** Bone density**	0.008	0.062	0.084	0.100	0.136	0.0262	<0.001	0.368
** Bone ash**	−0.009	0.057	0.082	0.098	0.139	0.0156	<0.001	0.360
** Bone ash**	−0.029	0.042	0.108	0.117	0.148	0.0185	<0.001	0.113
**Metacarpal**								
** Bone density**	0.046	0.082	0.102	0.107	0.177	0.0225	<0.001	0.366
** Bone ash**	0.061	0.076	0.111	0.155	0.172	0.0243	<0.001	0.126
** Bone ash**	−0.006	0.082	0.105	0.125	0.178	0.0293	<0.001	0.391
**Average** ^d^								
** Bone density**	0.016	0.068	0.082	0.098	0.154	0.0231	<0.001	0.484
** Bone ash**	0.015	0.066	0.092	0.130	0.152	0.0151	<0.001	0.075
** Bone ash**	−0.024	0.054	0.099	0.115	0.147	0.0175	<0.001	0.145

a Available P release by Microtech phytase was calculated using a standard response curve that utilized the inorganic P diets. Standard response curves were developed using marginal aP intake (i.e., dietary aP% minus 0.11% [the aP in the basal diet] multiplied by ADFI) as the predictor variable for each response criterion. Marginal aP intake equivalence was solved for each replicate pen fed a specific phytase by substituting the response variable in each standard response equation with the measured value. Using the pen ADFI, aP intake was then converted to marginal aP release.

b Microtech phytase, VTR Biotech, Guagdong, China.

c One pig per pen (8 pigs per treatment) was euthanized and the right fibula, right 10th rib, and right third metacarpal were collected to determine bone density, bone ash weight, and percentage bone ash. After cleaning, bones were submerged in ultra-purified water under vacuum for 4 h. Weights were then collected, and bone density calculated. For bone ash, bones were placed in a drying oven at 105°C for 7 d and then ashed in a muffle furnace at 600°C for 24 h.

d Average aP release values generated using data from the right fibula, rib, and metacarpal.

Bone density, bone ash weight, and percentage bone ash of the fibula, rib, metacarpal and their average increased (linear, *P ≤ *0.004) with increasing aP and STTD P from inorganic P and increasing phytase (Table 4). As a result, the calculated percentage aP release from Microtech phytase based on different bone response criteria followed a significant linear (*P ≤ *0.002) pattern.

Percentage fecal DM, N, and P were not influenced by increasing aP and STTD P from inorganic P, whereas increasing phytase decreased fecal N (quadratic, *P = *0.015) and fecal P (linear, *P = *0.009) percentage (Table 4). Dry matter and N digestibility decreased (quadratic, *P ≤ *0.049) with increasing aP and STTD P from inorganic P, while P digestibility increased (linear, *P < *0.001) with increasing aP and STTD P from inorganic P and increasing phytase.

**Fig. 1.**
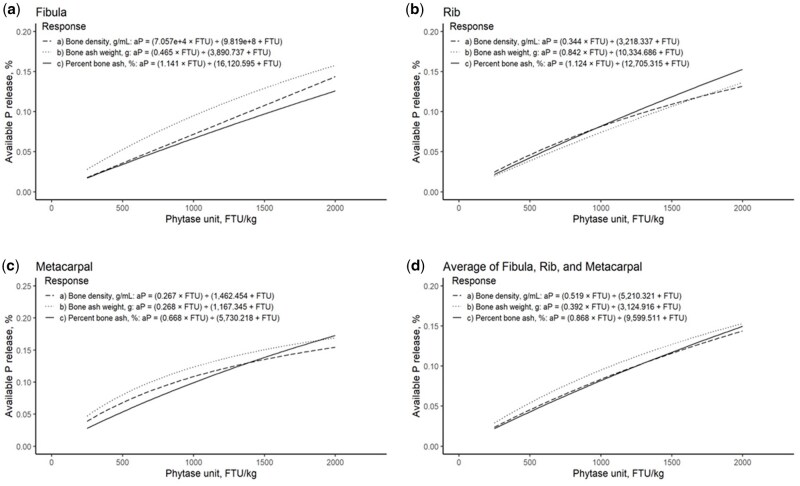
Available P release curves of Microtech phytase for bone density, bone ash weight, and percent bone ash for a) right fibula, b) right 10th rib, c) right third metacarpal, and d) the average of all three bones.

The aP release curves of Microtech phytase for bone density, bone ash weight and percent bone ash for the right fibula, right 10th rib, right third metacarpal, and the average of all three bones were modelled following Equation 3 (Fig. 1a–d). The aP release based on average bone density (Equation 4), bone ash weight (Equation 5) and percent bone ash (Equation 6) of the 3 bones, respectively are:


(4) aP release, %=(0.519×FTU/kg) ÷ (5210.321+FTU/kg).

(5) Bone ash weight aP release, %=(0.392×FTU/kg) ÷ (3124.916+FTU/kg).

(6) Bone ash (%) aP release, %=(0.868×FTU/kg) ÷ (9599.511+FTU/kg).

**Fig. 2.**
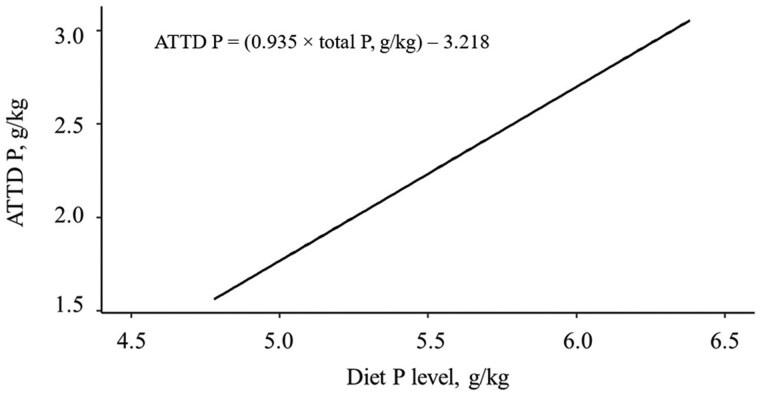
Regression of ATTD P (g/kg feed) against dietary total P concentration (g/kg feed) in inorganic P diets to estimate the TTTD coefficient of P in MCP. The regression equation was: ATTD P = (0.935 × total P) − 3.218 (*r*^2^ = 0.737) with the slope representing the TTTD of P of MCP and the *y*-intercept representing the total endogenous losses. The TTTD of P of MCP was used as multiplier to convert aP release to digestible P release.

**Fig. 3.**
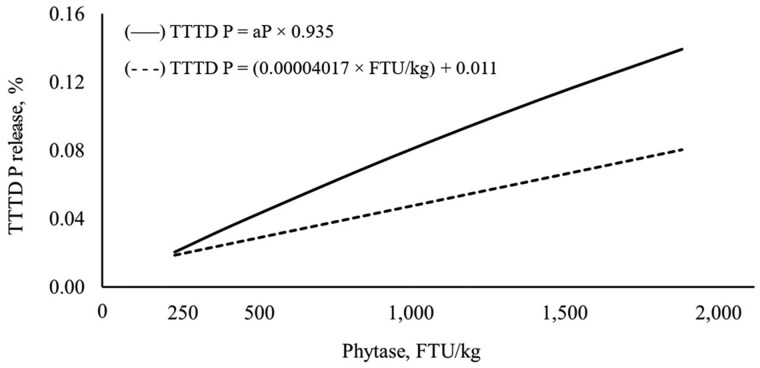
Standardized total tract digestible P release with supplemental Microtech phytase: approach 1: TTTD P = aP × 0.935 (—–) and approach 2: TTTD P = (4.017e^−5^ × FTU) + 0.011, *r*^2^ = 0.236 (-----).

For TTTD P release curve of Microtech phytase, two approaches adapted from Zhai et al. (2023) were used. In both approaches, the TTTD P release increased (linear, *P < *0.001) with increasing phytase (Table 6). The first approach resulted in greater digestible P release values at 0 to 0.14% TTTD P than the second approach at 0.02% to 0.09% TTTD P. For the first approach, the regression equation was: ATTD P = (0.935 × total P) − 3.218 (*r*^2^ = 0.737) with the slope representing the TTTD of P of monocalcium phosphate and the *y*-intercept representing the total endogenous losses (Fig. 2). The TTTD of P of monocalcium phosphate was used as multiplier to convert aP release to digestible P release (Approach 1: TTTD P = aP × 0.935). For the second approach, the STTD P release curve of Microtech phytase as TTTD P = (0.00004017 × FTU/kg) + 0.011 (Fig. 3).

**Table 6.** Calculated TTTD P release values based on 2 approaches demonstrated by Zhai et al. (2023).

Item	Phytase, FTU/kg^a^					SEM	*P* =	
	250	500	1000	1500	2000		Linear	Quadratic
**Approach 1** ^b^								
** TTTD P, %**	−0.022	0.051	0.092	0.107	0.137	0.0163	<0.001	0.144
**Approach 2** ^c^								
** TTTD P, %**	0.022	0.038	0.038	0.081	0.090	0.0179	<0.001	0.849

a Microtech phytase, VTR Biotech, Guagdong, China.

b The aP release values based on bone ash weight of the average of the 3 bones (Table 5) were transformed to its equivalent TTTD P values by multiplying to the TTTD coefficient of P in monocalcium phosphate of 0.935.

c The TTTD P release by Microtech phytase was calculated for each pen by subtracting the average of the TTTD P in the control diet (no phytase) from the TTTD P in each phytase-containing diet.

## Discussion

Microbial phytase is an enzyme used to release the phytate bound P and its complexes in plant-based raw materials. Phytase reduces the antinutritional effect of phytate through dephosphorylation of insoluble phytic acid in grains and oilseeds into orthophosphate and inositol phosphate, allowing improved digestion of P, Ca, amino acids, and energy, thereby improving animal performance while reducing P excretion (Adeola and Cowieson 2011; Dersjant‐Li et al. 2015). Several exogenous phytases are commercially available and their efficacy varies, thus knowing the efficacy of P release from novel phytase is essential for its effective use in diet formulation (Becker et al. 2021; Kim et al. 2024). Undervalued dietary P-release from phytase results in unnecessary diet cost and a negative environmental impact, whereas over estimation of P-release from phytase may compromise animal growth and bone mineralization. In this experiment, Microtech phytase developed by VTR Biotech (Guangdong, China) is an *E.* coli-derived 6-phytase that is expressed in *Pichia pastoris*. Internal evaluation of Microtech phytase showed that it is stable after feed conditioning and pelleting (75°C to 80°C) and is stable at a pH range of 3.0 to 5.5.

Unlike previous studies that evaluated the effect of Microtech phytase in nursery pig diets, the current study incorporated canola meal to achieve a higher dietary phytate P level of 0.32%, compared to 0.24% typically found in corn–soybean meal-based diets. This adjustment was made to ensure an adequate supply of substrate for phytase activity. Canola meal contains 0.65% phytate P and 1.08% total P, whereas soybean meal contains 0.38% phytate P and 0.71% total P (NRC 2012).

An earlier evaluation of Microtech phytase was conducted by Flohr et al. (2015) where 15 to 28 kg nursery pigs were either fed an increasing dietary aP from 0.12% to 0.24% aP using inorganic P or increasing dietary phytase from 155 to 780 FTU/kg using from Microtech phytase. A linear increase in final BW and ADG and a quadratic increase in G:F with increasing phytase in the diet were observed by Flohr et al. (2015) demonstrating a potential further benefit beyond the highest level evaluated of 780 FTU/kg. In the current study, the inclusion was increased above the levels of Flohr et al. (2015) and observed final BW, ADG, ADFI and G:F were numerically greater at the highest inclusion rate of 2000 FTU/kg although quadratic responses were observed. The greatest magnitude of improvement in final BW and ADG was observed with the first 500 FTU/kg inclusion with smaller incremental improvement through 2000 FTU, whereas Flohr et al. (2015) observed greater magnitude of improvement in ADG between 465 to 780 FTU/kg.

Besides the improvement in growth performance, phytase in the diet al.ows for reduced use of inorganic P and decreases P excretion. In the current study, fecal P linearly decreased with increasing phytase in the diet, resulting in a linear increase in P digestibility up to the highest phytase level of 2000 FTU/kg. This is in agreement with Symeou et al. (2014) who modeled P digestibility, retention and excretion from added dietary phytase and observed that the responses were curvilinear and P digestibility reached a maximum at 2500 FTU/kg. In contrast to P digestibility, DM and N digestibility were not influenced by phytase levels. In a series of studies conducted by Liao et al. (2005), supplementation of phytase at 500 and 1000 FTU/kg improved N digestibility in wheat-soybean meal- and wheat-soybean meal-canola meal based diets, but not in corn-soybean meal and barley-peas-canola meal-based diets which may suggest that the response depends on diet composition. Apart from the fecal P and P digestibility response to increasing phytase, it is important to note that the increasing aP in the inorganic P diets, thus increasing P intake, increased P digestibility without significantly increasing fecal P, suggesting the extra P consumed was retained. In contrast, Ekpe et al. (2002) observed a linear increase in fecal P with increased dietary P intake. The difference in results is because P levels in diets of the current study were intentionally below the pig’s P requirement to generate a standard curve.

As P is needed to maximize bone strength and mineralization, these parameters have been included in numerous phytase studies (Becker et al. 2021; Gaffield et al. 2023; Kim et al. 2024). Consistent with these studies, multiple bones and bone criteria were used in the present study as numerous factors may influence the sensitivity of a bone to dietary P. For example, Williams et al. (2022) concluded that in 12-kg pigs, the second rib and fibula were the most sensitive indicators of P status in bone mineralization. In contrast, Crenshaw et al. (1981) reported that the femur, humerus, and rib were the most responsive to P in younger pigs. Congruent with the findings of Flohr et al. (2015), linear increases in bone ash weight and bone ash percentage were observed in the present study. Additionally, bone density, which is another measure of bone mineralization, linearly increased with increasing phytase in the diet. This suggests that a greater inclusion of Microtech phytase (i.e., >2000 FTU/kg) may further increase these bone characteristics.

Using the responses of bone criteria and fecal P to increasing phytase in the diet, the aP and TTTD P release values were determined. Phosphorus release is often evaluated using aP release curves as it represents the amount of P that is digested, absorbed, and available for utilization in bone mineralization (Becker et al. 2021; Gaffield et al. 2023; Kim et al. 2024). In this approach, the aP release by phytase is calculated by comparing it with standard mineral P source (e.g. monocalcium phosphate) to achieve the same response of a sensitive indicator such as bone ash (Dersjant-Li et al. 2019), although growth performance is also used. The NRC (2012) P requirement estimates are represented as digestible P. Therefore, it is important to establish both aP and digestible P release by phytase. Different aP to digestible P conversions have been used. For example, available P can be converted to STTD P using a factor of 0.883 which is based on assumed P relative bioavailability of 100% and an actual STTD P of 88.3% of monocalcium phosphate (NRC 1998, 2012). This conversion has been challenged by relevant studies because aP and digestible P are different in several aspects including that digestible P is point-of-time measurement while aP reflects a cumulative effect of P on bone mineralization (Zhai et al. 2022, 2023). Moreover, digestible P is reflective of the digested and absorbed P in a diet with specific ingredient composition; whereas aP accounts for the P balance in animals. In the current study, both aP and digestible P (expressed as TTTD P) of Microtech phytase were determined and TTTD P was determined following the two approaches described by Zhai et al. (2023). Compared to ATTD P, TTTD P corrects the digestibility coefficient of P for the total endogenous losses (She et al. 2017). In this study, total endogenous losses were estimated using regression analysis as described by Pettey et al. (2006).

The aP release values for phytase depend on response variables as these have different sensitivity to dietary P supply. In the current study and similar to findings of Zhai et al. (2023), the aP release values based on growth performance were higher than the bone criteria and this can be explained by the dependence of soft tissue growth on P and the extra-phosphoric effect of phytase beyond P nutrition (Lu et al. 2019; Lautrou et al. 2020). However, contrary to Zhai et al. (2023), a quadratic trend in calculated aP release values based on ADG and G:F but a linear trend in calculated aP release based on bone criteria were observed in the present study. This supports the knowledge that bone mineralization is a more sensitive parameter for dietary P-related studies than other parameters (Wang et al. 2021) and thus provides evidence that it provides a better estimation of aP release. Furthermore, the aP release based on bone ash weight was greater than aP release based on bone density and bone ash percent. Bone criteria based on weight generally give greater response to supplementation of monocalcium phosphate or phytase than bone criteria in percentages because bone size adds another dimension of difference. As growth in size might obscure the improvement in mineral deposition, bone ash percentage may provide a more accurate measure of aP release from phytase as it independent of bone size (Gaffield et al. 2023). Additionally, higher aP release was observed for third metacarpal than fibula and 10th rib. This is in contrast with the findings of Kim et al. (2024) who observed greater aP release with 10th rib than third metacarpal and fibula. The differences among studies suggest that the average of the three bones may provide a more robust estimate of the aP release.

For the TTTD P, similar to Zhai et al. (2023), the current study used two approaches. The first approach that utilized the TTTD P of monocalcium phosphate of 0.935 (the slope of the linear regression of ATTD P against the formulated dietary P concentration) to convert aP to TTTD P release by phytase generated greater values than the second approach that utilized actual TTTD P of the phytase diets, and the difference was greater at higher phytase levels. For example, at 500 FTU/kg, the TTTD P release based on approaches 1 and 2 are 0.05% and 0.04%, respectively, whereas at 2000 FTU/kg, the TTTD P release based on approaches 1 and 2 are 0.14% and 0.09%, respectively. The difference can be explained by the different response criteria used. The first approach used aP that was measured using bone mineralization which is a more sensitive response parameter than P digestibility which was used in the second approach. This paper used the second approach to estimate TTTD P release because it directly measured the improvement in digestibility by phytase.

In summary, this study provided the aP and TTTD P release values for Microtech phytase in nursery pigs weighing 10 to 22 kg nursery pigs when fed at levels between 250 to 2000 FTU/kg. The aP and TTTD P release of Microtech phytase depends on the bone characteristic measured, type of bone used, and the methodology used to estimate digestibility of P. The aP release curve based on the average percent bone ash of the three bones and TTTD P release curve based on directly improved digestibility for Microtech phytase are: aP release, % = (0.868 × FTU/kg) ÷ (9599.511 + FTU/kg) and TTTD P release, % = (0.00004017 × FTU/kg) + 0.011, respectively.

## List of abbreviations:

ADFI: average daily feed intake
ADG: average daily gain
aP: available P
ATTD P: apparent total tract digestible P
BW: body weight
DM: dry matter
FTU: phytase unit
G:F: gain to feed ratio
NE: net energy
STTD P: standardized total tract digestible P
TTTD P: true total tract digestible P
